# Retraction: Root foraging of birch and larch in heterogeneous soil nutrient patches under water deficit

**DOI:** 10.1371/journal.pone.0299548

**Published:** 2024-02-22

**Authors:** The PLOS ONE Editors

The *PLOS ONE* Editors retract this article as it was identified as a submission for which we have concerns over similarity to work by another group and regarding the integrity of peer review.

LT, RF and SG did not agree with the retraction. HS either did not respond directly or could not be reached.
